# Impact of Phosphatemia Variability in Neurological Outcomes in Patients With Spontaneous Subarachnoid Hemorrhage

**DOI:** 10.7759/cureus.18257

**Published:** 2021-09-24

**Authors:** Rita Varudo, Ana Marta Mota, Eduarda Pereira, Celeste Dias

**Affiliations:** 1 Intensive Care Department, Hospital Garcia de Orta, Almada, PRT; 2 Intensive Care Department, Hospital Central Funchal, Funchal, PRT; 3 Neurocritical Care Unit, Intensive Care Department, Hospital São João, Porto, PRT

**Keywords:** subarachnoid hemorrhage, phosphate, hypophosphatemia, glasgow coma scale, glasgow outcome scale

## Abstract

Introduction: Electrolyte disturbances, such as dysnatremia, hypokalemia, and hypomagnesemia, are frequently observed during acute spontaneous subarachnoid hemorrhage (sSAH). However, there are limited data concerning hypophosphatemia.

Objective: To analyze the frequency of phosphate (Pi) disturbances in sSAH patients and assess their influence on neurological outcomes compared with that in patients without sSAH.

Methods: We conducted a retrospective study of patients with sSAH admitted to a neurocritical care unit in two years. We also included nonneurocritical patients admitted to a general intensive care unit (ICU). Serum Pi levels and daily Pi repletion data were collected during the first 10 days after admission. The primary endpoint was neurologic outcome using the Glasgow Outcome Scale at six months (GOS-6M) and the Glasgow Coma Scale at ICU discharge (GCS-ICUd). The effect of phosphatemia variability on mortality and ICU length of stay (ICU-LOS) was also analyzed.

Results: Patients with sSAH had lower mean Pi level and median Pi dose repletion than that of nonneurocritical patients (3.1 ± 0.4 vs. 3.9 ± 1.3, p < 0.001). In the sSAH group, patients with hypophosphatemia had lower GCS-ICUd (12 ± 3.3 vs. 14 ± 2.4). Also, GOS-6M was lower in patients with hypophosphatemia but was not statistically significant (p = 0.09). By contrast, a higher mean Pi level in nonneurocritical patients was significantly associated with higher ICU mortality (4.8 ± 1.6 mg/dL vs. 3.6 ± 1.0 mg/dL, p = 0.003) and higher ICU-LOS (r = 0.231, p = 0.028). In the sSAH group, we found the opposite. In a multivariate analysis of the sSAH group, the increase in the Pi level was associated with higher GCS-ICUd (unstandardized coefficient in multiple linear regression [B] 1.79; 95% CI 0.43-3.15). The opposite was found in nonneurocritical patients. A Pi concentration higher than 2.5 mg/dL was associated with a better GCS-ICUd. We also found that creatinine, urea, chloride, need for Pi substitution, therapy intensity level, and pH were independent predictors of the mean Pi level during ICU stay in the sSAH group.

Conclusions: Patients with sSAH had lower mean Pi levels and required significantly higher daily Pi replacement compared with those of nonneurocritical patients. Since hypophosphatemia may be associated with poor neurological outcomes, patients with sSAH need cautious phosphate repletion.

## Introduction

Spontaneous subarachnoid hemorrhage (sSAH) can occur because of the rupture of an aneurysm or arteriovenous malformation, hypertension, or an unknown cause [1]. In Europe, approximately 36,000 new cases of subarachnoid hemorrhage (SAH) occur per year. Despite improvements in surgical, radiological, and medical treatment, the rupture of an aneurysm is still associated with a high incidence of mortality and severe long-term disability [2]. Outcomes after SAH depend mainly on the initial severity of the hemorrhage. Nonneurological complications can also contribute to a worse prognosis [3,4].

Electrolyte disturbances are common in patients in the intensive care unit (ICU). Intracranial changes are associated with ionic disorders; hence, they are important in neurocritical patients [3]. Dysnatremia, hypomagnesia, and hypokalemia frequently occur in the acute period after SAH, but studies on electrolyte disturbances in SAH have controversial results [3,5].

Phosphate (Pi) is a major intracellular anion that plays an important role in many biochemical pathways relating to normal physiological functions, especially maintaining muscle tone. Hypophosphatemia is associated with muscle weakness, including weakness of respiratory muscles, and respiratory infection. Low Pi may also be associated with decreased cardiac output and ventricular tachycardia after myocardial infarction [6].

Hypophosphatemia is relatively common in critically ill patients with an incidence of 20% [7]. There are few studies regarding hypophosphatemia after a neurological insult, particularly in patients with sSAH or other intracranial hemorrhages. The truth is that many symptoms of hypophosphatemia, such as altered mental status, seizures, and hemodynamic instability are common in this patient population; therefore, its correction can be relevant in the sSAH approach [7].

The primary endpoint of this study is to assess the influence of Pi disturbances and repletion in neurological outcomes in patients with sSAH and compare them with those in nonneurocritical patients. The secondary endpoint is to determine the effect of phosphatemia variability on mortality and ICU length of stay. This research was previously presented as a meeting abstract at the European Society of Intensive Care Medicine LIVES 2020 on December 7, 2020.

## Materials and methods

Study population and clinical management

**Figure 1:**
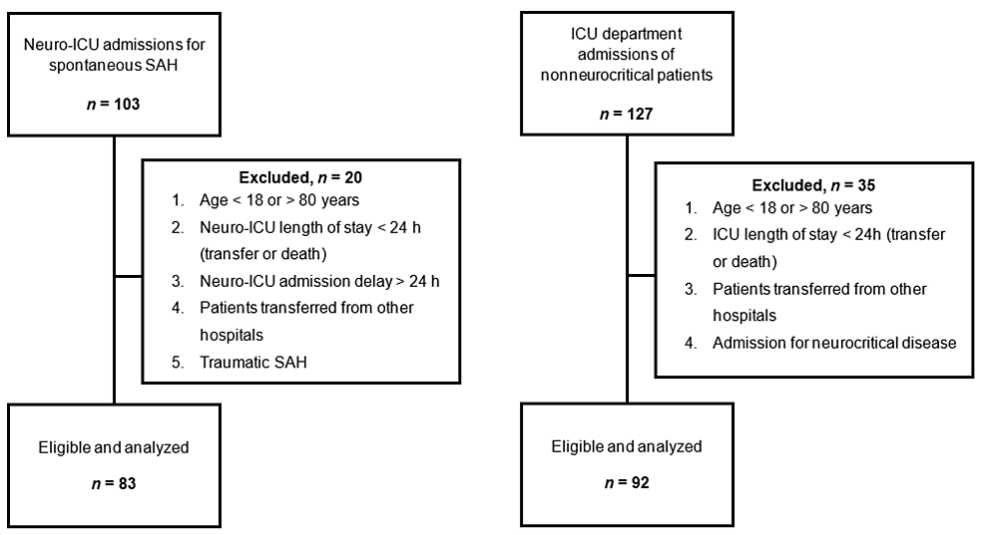
Study flowchart ICU - intensive care unit; SAH - subarachnoid hemorrhage.

This single-center, retrospective, observational study included consecutive patients with an sSAH diagnosis (including all patients with nontraumatic SAH) admitted to a neurocritical care unit (Neuro-ICU) of a Portuguese University Hospital between January 2017 and December 2018. We also included a group of nonneurocritical patients (to evaluate differences in Pi levels during ICU stay) admitted to an ICU at the same center and over the same period, who were randomly selected, with the same exclusion criteria. The exclusion criteria were age under 18 or over 80 years, ICU length of stay less than 24 hours (transferred or death), transfer from another institution, and traumatic SAH. The flowchart of this study is presented in Figure 1.

This study was approved by the Ethics Committee of São João University Hospital Center (494/20). This retrospective study enrolled patients after ICU discharge and was not related to endovascular or surgical treatment; therefore, consent was not required.

Patient characteristics, including age, gender, and medical history, were recorded. The level of consciousness on hospital admission (or before sedation, if earlier) was assessed with the Glasgow Coma Scale (GCS) [8].

The diagnosis of SAH was established by cranioencephalic computed tomography on admission. SAH was then classified using the Fisher and Hunt & Hess Scales [9,10]. All patients were treated according to standard neurocritical care consistent with the latest guidelines during their ICU stay [11-13]. Hemorrhage was treated using an endovascular or surgical procedure in the early phase of care. The neurosurgeon and neuroradiologist determined the timing and type of management, independently of the study protocol. Patients with aneurysmal bleeding were treated with intravenous nimodipine, avoiding hypovolemia to minimize the risk of vasospasm. Excessive volume loading was also avoided. Mean arterial blood pressure was targeted individually between 60 and 80 mmHg or even above these values if delayed cerebral ischemia was suspected, as long as the aneurysm was secured. Intracranial pressure was monitored, targeting the mean arterial blood pressure to maintain optimal cerebral perfusion pressure using the pressure reactivity index. The administration of vasoactive drugs and fluids was guided by the patient's hemodynamics, optimal cerebral perfusion pressure, and tissue perfusion parameters (lactate and arterial blood gas analysis). Hemoglobin, blood glucose, and electrolyte disturbances were corrected to obtain normal levels. Normal or mild hypocarbia was targeted individually. Data concerning ICU scores were retrieved from a retrospectively-collected electronic patient database (B-simple) in which the Acute Physiology and Chronic Health Evaluation II (APACHE II) score [14] and the Simplified Acute Physiology Score II (SAPS II) [15] are recorded in the first 24 hours after admission. There was no strict protocol regarding intravenous phosphate substitution therapy. The attending physician made decisions regarding therapy. However, the typical approach was that if there was associated hypophosphatemia, monopotassium phosphate or dipotassium phosphate was administered; otherwise, the correction was made using sodium glycerophosphate.

Each patient's participation in the study began on the day of ICU admission and lasted for 10 days, unless the patient died or was transferred to another hospital. Arterial blood samples for measurement of Pi were taken on patient admission and subsequently once every day. Analyses were performed in an accredited central laboratory. In the case of blood sample analyses twice daily, the first measurements were recorded and analyzed.

From the database, maximal, minimal, and mean Pi concentrations during ICU stay were obtained for each patient, indicating the highest or lowest value recorded. Hypophosphatemia was defined as Pi < 2.5 mg/dL; normophosphatemia was Pi = 2.5-4.5 mg/dL; and hyperphosphatemia was as Pi > 4.5 mg/dL [16]. The duration of hypophosphatemia was calculated as the number of days Pi was less than 2.5 mg/dL.

Daily diuresis and the cumulative fluid balance were calculated and recorded. Enteral nutrition (EN) was also evaluated and recorded as the percentage of ICU days that each patient was on EN. Daily blood glucose, urea, creatinine, hematocrit, and therapy intensity level (TIL) scale for intracranial pressure management were also recorded. Blood pH, partial pressure of carbon dioxide (pCO2), and partial pressure of oxygen (pO2) were obtained from blood gas samples. Then, the influence of these variables in Pi level variations was analyzed.

Phosphate substitution therapy administered as monopotassium phosphate, dipotassium phosphate, and sodium glycerophosphate was recorded, and the total daily repletion was calculated.

Outcomes

Neurological outcomes were assessed using GCS at ICU discharge (GCS-ICUd) and the Glasgow Outcome Scale six months after discharge (GOS-6M) [17]. Glasgow Outcome Scale (GOS) was dichotomized as good (GOS four-five: no disability to moderate disability) or poor (GOS one-three: severe disability, vegetative state, death). We used GOS-6M in the sSAH group because it is validated only for brain injury.

The ICU-LOS and ICU mortality were also recorded. Data were collected during clinical process review.

Statistical analysis

Continuous data were expressed as means with standard deviations (mean ± SD) or medians and interquartile ranges (med ± IQR) depending on the underlying data distribution. Categorical data were reported as proportions. Univariate analysis was performed to determine the effect of Pi variability in outcomes of patients with and without sSAH. Continuous variables were analyzed using Student's t test or the Mann-Whitney U test, the latter for nonnormal data distribution. The chi-squared test or Fisher's exact test was used for categorical variables. Pearson's correlation coefficient was used to assess continuous variables. Multiple linear regressions assessed the effect of phosphatemia on neurological outcomes and determined the best model for the prediction of GCS-ICUd and GOS-6M. Multivariate analysis was performed to identify independent predictors of phosphate variability. The receiver operating characteristic (ROC) curve and area under the curve (AUC) were calculated to identify a cutoff point for the Pi level associated with better GCS at ICU discharge. All analyses were performed using SPSS Statistics, version 22.0 (IBM Corp., Armonk, NY). Two-tailed p-values were reported, and a p-value < 0.05 was considered statistically significant.

## Results

Characterization of the study population

During the two-year study period, 83 patients with sSAH were treated in the Neuro-ICU, and 92 nonneurocritical patients admitted to the same ICU department were evaluated (Figure 1). The main characteristics of the two groups are described in Table 1.

**Table 1:** Characterization of groups. APACHE II - Acute Physiology and Chronic Health Evaluation II; SAPS II - Simplified Acute Physiology Score II; GCS - Glasgow Coma Scale; TIL - Therapy intensity level; EN - Enteral nutrition; paO2 - Partial pressure of oxygen; FiO2 - Fraction of inspired oxygen; pCO2 - partial pressure of carbon dioxide; ICU - Intensive Care Unit; IMV - invasive mechanical ventilation; GOS - Glasgow Outcome Scale; IQR - Interquartile range * To evaluate the influence of enteral nutrition, we calculate the percentage of days of ICU stay that each patient had with enteral nutrition.

Characteristic	sSAH group (n = 83)	Nonneurocritical group (n = 92)	p-value
Age, years – mean ± SD	56.4 ± 12.1	63.7 ± 13.1	<0.001
Male sex, n (%)	47 (56.6)	39 (42.4)	0.06
APACHE II, mean ± SD	14.0 ± 6.4	23.7 ± 9.2	<0.001
SAPS II, mean ± SD	30.5 ± 14.5	50.6 ± 18.7	<0.001
Fisher grade, n (%)			
I	4 (4.8)	-	-
II	9 (10.8)	-	-
III	14 (16.9)	-	-
IV	56 (67.5)	-	-
Hunt-Hess score, n (%)			
I	11 (13.3)	-	-
II	39 (47.0)	-	-
III	12 (14.5)	-	-
IV	10 (12.0)	-	-
V	11 (13.3)	-	-
GCS at admission, med (IQR)	14 (5)	14 (6)	0.699
TIL, med (IQR)	0.3 (1.2)	-	-
EN, % days*, med (IQR)	80 (10)	50 (45)	<0.001
Hematocrit, %, mean ± SD	34.3 ± 3.8	31.2 ± 5.7	<0.001
Blood glucose, mg/dL, med (IQR))	141.4 (46.9)	147.6 (34.0)	0.094
Blood urea, mg/dL, med (IQR)	29.8 (12.7)	60.5 (63.4)	<0.001
Blood creatinine, mg/dL, med (IQR)	0.5 (0.3)	1.1 (1.3)	<0.001
Phosphate level, mg/dL, mean ± SD	3.10 ± 0.44	3.88 ± 1.28	<0.001
Blood sodium, mmol/L, med (IQR)	140.0 (5.6)	141.0 (5.0)	0.565
Blood potassium, mmol/L, mean ± SD	3.9 ± 0.2	4.0 ± 0.4	0.105
Blood Chloride, mmol/L, med (IQR)	107.5 (5.2)	106.9 (8.8)	0.329
Fluid balance, L/day, med (IQR)	0.5 (0.8)	0.4 (0.7)	0.900
Urine output, L/day, med (IQR)	2.6 (0.7)	1.8 (1.7)	<0.001
PaO_2_/FiO_2_ ratio, mean ± SD	354.6 ± 88.5	282.9 ± 83.5	<0.001
pO_2_,in mmHg, med (IQR)	106.4 (54.5)	98.5 (26.8)	0.018
pCO_2_,_, _in mmHg, mean ± SD	36.6 ± 4.5	39.1 ± 6.3	0.005
Need for IMV, days, med (IQR)	1.0 (18)	0.0 (0)	<0.001
ICU length of stay, days, med (IQR)	17.0 (15)	5.5 (9)	<0.001
ICU mortality, n (%)	5 (6)	23 (25)	0.001
Hospital mortality, n (%)	5 (6)	32 (34.8)	<0.001
GCS at ICU discharge, med (IQR)	15 (1)	15 (12)	0.041
GOS at 6 months, med (IQR)	5 (1)	-	-

Phosphate levels, substitutions, and outcomes

There was no significant difference between the mean Pi level and the age or gender in the two groups. Higher APACHE II and SAPS II scores were associated with a higher mean Pi level in nonneurocritical patients but not in the sSAH group (r = 0.31, p = 0.003 and r = 0.28, p = 0.008, respectively).

There were 611 phosphate measurements in the sSAH group (mean of 7.3 measurements per patient) with a minimum value of 0.9 mg/dL and a maximum value of 6.9 mg/dL. In the nonneurocritical group, 496 Pi measurements were performed (mean of 5.3 measurements per patient) with a minimum value of 0.9 mg/dL and a maximum value of 10.7 mg/dL. The mean Pi value was significantly lower in the sSAH group (3.10 ± 0.44 vs. 3.88 ± 1.28, p < 0.001).

**Figure 2:**
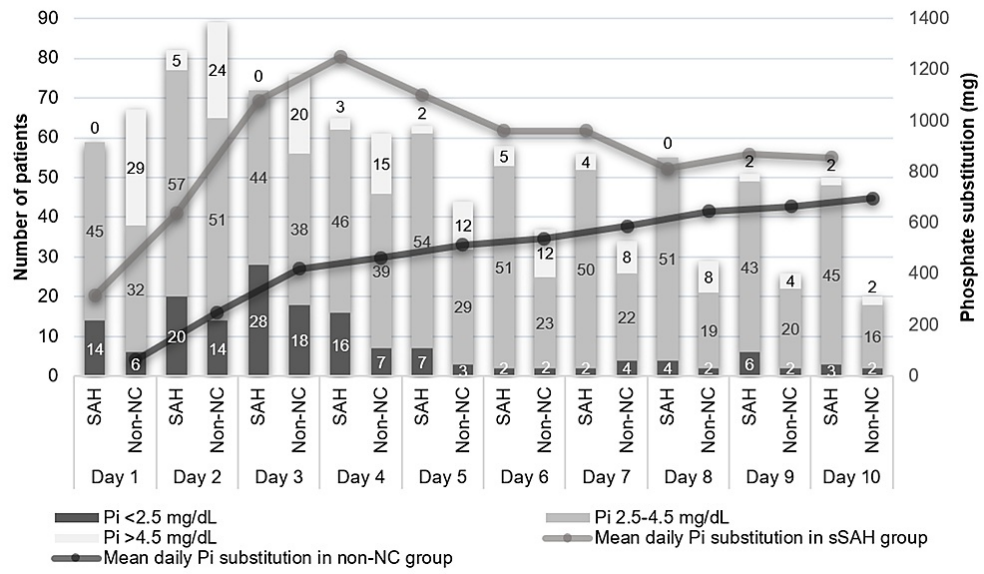
Daily phosphate substitution and the number of patients with hypo-, normo- and hyperphosphatemia in the sSAH and the nonneurocritical groups. sSAH - spontaneous subarachnoid hemorrhage; Pi - phosphate

Phosphatemia was classified as hypo-, normo- and hyperphosphatemia. The number of patients in each category in the first 10 days in the two groups is shown in Figure 2. Hypophosphatemia was more frequent in sSAH patients, mostly in the first four days. We detected at least one episode of hypophosphatemia in 52 patients (63%) with sSAH and 35 patients (38%) in the nonneurocritical group (p = 0.001).

Hyperphosphatemia was detected in 17 patients (21%) from the sSAH group and 52 (57%) from the nonneurocritical group (p < 0.001). The duration and timing of the first onset of hypophosphatemia are illustrated in Table 2. Hypophosphatemia at admission was more frequent in the sSAH group (n = 14 vs. n = 6, p = 0.024). The maximum number of days of hypophosphatemia was six in the sSAH group and four in nonneurocritical patients. The median dose of Pi replacement was also higher in the sSAH group [696 (1045) mg vs. 100 (284) mg, p < 0.001].

**Table 2:** Comparison of phosphate levels in the two groups. ICU - intensive care unit; IQR - Interquartile range * 24 missing values in the sSAH group and 25 in the nonneurocritical group

	sSAH group (n = 83)	Nonneurocritical group (n = 92)	p-value
Phosphate level, mg/dL			
Minimum, med (IQR)	2.30 (0.80)	2.60 (1.40)	0.001
Maximum, med (IQR)	3.90 (0.80)	4.75 (2.05)	<0.001
Mean during ICU stay, mean ± SD	3.10 ± 0.44	3.88 ± 1.28	<0.001
Incidence of hypophosphatemia, n (%)	52 (62.7)	35 (38)	0.001
Incidence of hyperphosphatemia, n (%)	17 (20.5)	52 (56.5)	<0.001
Incidence of both hypophosphatemia and hyperphophatemia, n (%)	11 (13.3)	12 (13.0)	0.967
Timing of the first onset of hypophosphatemia (days after ICU admission), n (%)*			
0	14 (23.7)	6 (9.0)	0.024
1	12 (14.6)	6 (6.7)	0.093
2 or more	24 (32.4)	18 (22.2)	0.153
Number of days with hypophosphatemia, n (%)			
1	27 (32.5)	19 (20.7)	0.075
2	12 (14.5)	10 (10.9)	0.475
3 days or more	13 (15.7)	6 (6.5)	0.052

**Figure 3:**
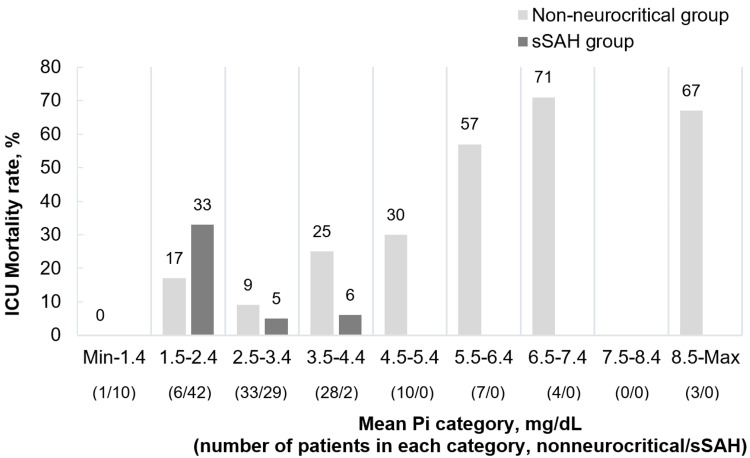
Crude ICU mortality according to the mean Pi concentration category during ICU stay. ICU - intensive care unit; sSAH - spontaneous subarachnoid hemorrhage; Pi - phosphate

Most of the patients who died in the nonneurocritical group had hyperphosphatemia at admission. A higher mean Pi level was associated with higher ICU mortality (4.8 ± 1.6 mg/dL vs. 3.6 ± 1.0 mg/dL, p = 0.003). By contrast, in the sSAH group, there was no significant difference, but the mean Pi level was lower in patients who died (2.8 mg/dL vs. 3.1 mg/dL, p = 0.086) (Figure 3). We also found that the Pi level at admission was significantly associated with ICU mortality in the nonneurocritical group (3.9 ± 1.3, p = 0.012).

**Figure 4:**
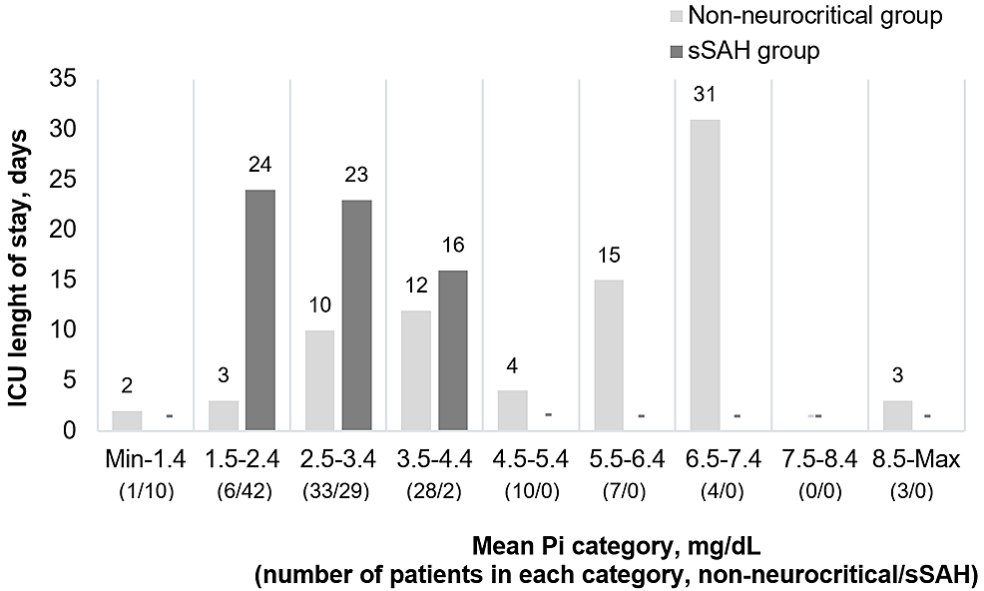
ICU length of stay according to the mean Pi concentration category during ICU stay. ICU - intensive care unit; sSAH - spontaneous subarachnoid hemorrhage; Pi - phosphate

The ICU-LOS was higher in nonneurocritical patients with a higher mean Pi level (r = 0.231, p = 0.028), and in the sSAH group, there was an inverse relation with no statistical significance (p = 0.595) (Figure 4).

Neurological outcomes were assessed based on GOS-6M and GCS-ICUd in the sSAH group and GCS-ICUd in the nonneurocritical group. In the sSAH group, patients with hypophosphatemia at admission had lower GOS-6M with no statistical significance (p = 0.09). sSAH patients with hypophosphatemia had lower mean GCS-ICUd than normophosphatemia patients (12 ± 3.3 vs. 14 ± 2.4, p = 0.004). In the nonneurocritical group, GCS-ICUd was lower with hypo- or hyperphosphatemia at admission (p = 0.009).

Hypophosphatemia during ICU stay, i.e. having at least one day with hypophosphatemia, was not significantly associated with neurological outcomes in the sSAH group (GCS-ICUd: p = 0.141; GOM-6M: p = 0.543).

When we compared the 10-day mean Pi level and GCS-ICUd, we found an inverse relation. There was a positive correlation between Pi levels and GCS-ICUd in the sSAH group (r = 0.23, p = 0.034) and a negative correlation in the nonneurocritical group (r = −0.36, p < 0.001). Thus, lower Pi levels were associated with lower GCS-ICUd in the sSAH group and higher GCS-ICUd in the nonneurocritical group.

There was no association between intravenous Pi substitution therapy and neurological outcomes (GOS-6M and GCS-ICUd) or ICU mortality in the sSAH group; however, there was a weak correlation with ICU-LOS (r = 0.24, p = 0.026). In the nonneurocritical group, there was a positive correlation between Pi substitution therapy and GCS-ICUd (r = 0.28, p = 0.007). In this group, patients who died during their ICU stay had a significantly lower Pi substitution level compared with ICU survivors [0 (111) vs. 158 (333), p = 0.004]. There was no association between Pi substitution and ICU-LOS.

All variables with a significant association with GCS-ICUd in the univariate analysis (p-value < 0.05) were considered for inclusion in the multivariate analysis. We included variables that significantly explained the variability in the dependent variable and determined the best model.

In the sSAH group, using GCS-ICUd as the dependent variable in the multiple linear regression analysis (Table 3), the mean Pi level during ICU stay (unstandardized coefficient in multiple linear regression [B] 1.79, 95% CI: 0.43-3.15), Pi substitution, days on EN, and SAPS II were significantly associated with GCS-ICUd (coefficient in multiple linear regression [R] = 0.79, R² = 0.62). We also concluded that a 1 mg/dL increase in Pi increases GCS-ICUd by 1.73 points. Thus, an increase in the Pi level seems to be associated with a better neurological outcome.

**Table 3:** Multiple linear regression model for GCS at ICU discharge in the sSAH group (R = 0.79, R² = 0.62). GCS - Glasgow Coma Scale at intensive care unit discharge; SAPS II - Simplified Acute Physiology Score II; pCO2 - partial pressure of carbon dioxide; B - Unstandardized coefficient in multiple linear regression; R - Coefficient in multiple linear regression

Independent variables	GCS-ICUd	
	B_nonstand_ (95% CI)	p-value
SAPS II	−0.10 (−0.15 to −0.06)	<0.001
Phosphate level, mg/dL	1.79 (0.43–3.15)	0.011
Phosphate substitution, mg	0.001 (0.000–0.001)	0.038
Hematocrit, %	−0.09 (−0.23–0.04)	0.174
Blood urea, mg/dL	−0.02 (−0.06–0.02)	0.241
Blood chloride, mmol/L	−0.11 (−0.22–0.001)	0.052
Urine output, L/day	−0.001 (−0.001–0.000)	0.060
Enteral nutrition, % of days	0.03 (0.01–0.06)	0.018
pCO_2_, mmHg	−0.02 (−0.06–0.02)	0.278

In the nonneurocritical group (Table 4), the Pi level had a significant influence on GCS-ICUd (R = 0.70, R² = 0.49), but in this case, an increase in Pi level seems to be associated with a poor neurological outcome; a 1 mg/dL increase in Pi decreases GCS-ICUd by 1.65 points (95% CI: −2.78 to −0.52).

**Table 4:** Multiple linear regression model for GCS at ICU discharge in the nonneurocritical group (R = 0.70, R² = 0.49). GCS - Glasgow Coma Scale at intensive care unit discharge; SAPS II - Simplified Acute Physiology Score II; pCO2 - partial pressure of carbon dioxide; B - Unstandardized coefficient in multiple linear regression; R - Coefficient in multiple linear regression

Independent variables	GCS-ICUd	
	B_nonstand_ (95% CI)	p-value
SAPS II	−0.07 (−0.12 to −0.01)	0.017
Phosphate level, mg/dL	−1.65 (−2.78 to −0.52)	0.005
Hematocrit, %	−0.25 (−0.41 to −0.09)	0.003
Blood urea, mg/dL	0.03 (0.01–0.05)	0.010
Blood potassium, mmol/L	−3.20 (−6.05 to −0.34)	0.029
Fluid balance, L/day	−0.99 (−1.85 to −0.12)	0.026
pCO_2_, mmHg	−0.15 (−0.31–0.01)	0.062

Mean Pi level predictors

Since the mean low Pi level was associated with a poor neurological outcome in our cohort of patients with sSAH, a multivariate analysis was conducted to identify predictors of the mean Pi level during ICU stay. We included variables that had a significant association with the mean Pi level in a univariate analysis to define the best predictor model (Table 5).

**Table 5:** Multiple linear regression models for predictors of the Pi level in the sSAH group (R = 0.73, R2 = 0.54). TIL - therapy intensity level; Pi - phosphate; B - Unstandardized coefficient in multiple linear regression; R - Coefficient in multiple linear regression

Independent variables	B (95% CI)	p-value
Fisher scale	−0.07 (−0.16–0.02)	0.118
TIL	−0.08 (−0.16 to −0.004)	0.039
Pi substitution (mg)	0.000 (0.000–0.000)	0.001
Creatinine (mg/dL)	−1.06 (−1.56 to −0.56)	<0.001
Urea (mg/dL)	0.02 (0.01–0.03)	<0.001
Chloride (mg/dL)	−0.02 (−0.04 to −0.01)	0.007
pH	0.39 (0.17−0.60)	0.001

Creatinine, urea, chloride blood levels, need for Pi substitution, TIL, and pH were significantly associated with the mean Pi level during ICU stay.

**Figure 5:**
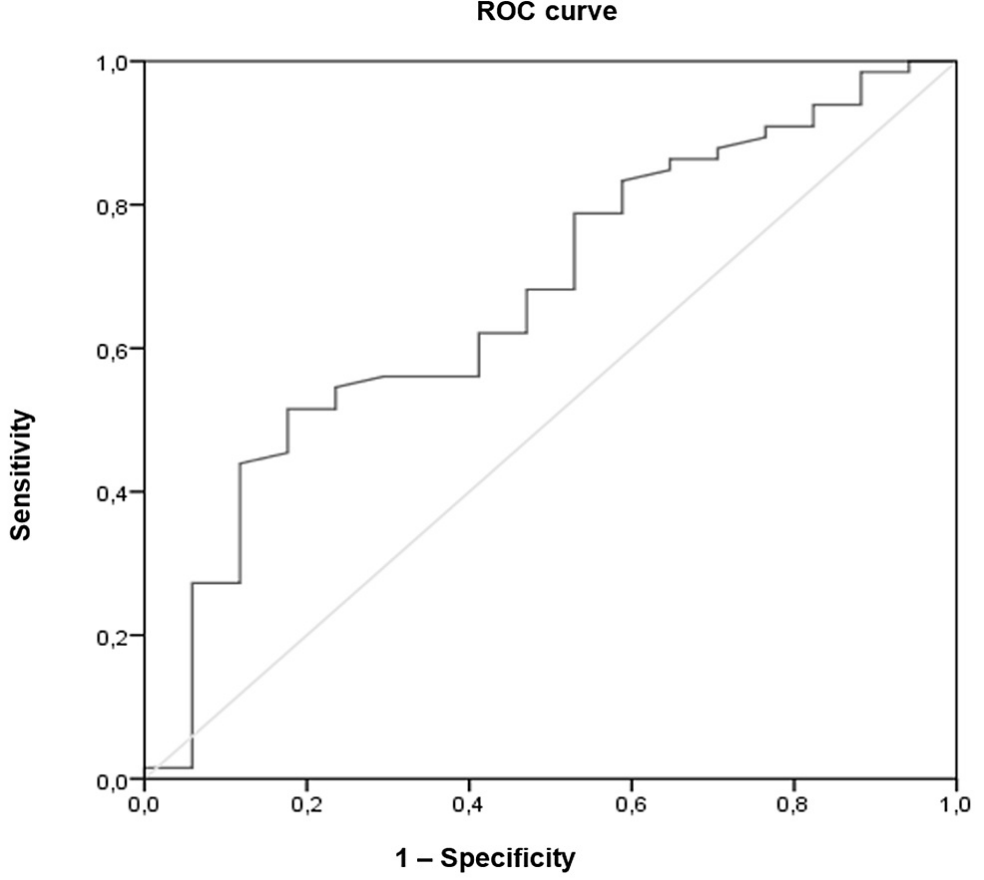
ROC for prediction of the phosphate level associated with a better neurological outcome (GCS 14-15) AUC = 0.673; p = 0.029. ROC - Receiver operating curve; AUC - Area under the curve

A ROC curve was performed to identify a mean Pi level cutoff point associated with GCS of 14-15 at ICU discharge. AUC was 0.673 (p = 0.029; CI 95%: 0.53-0.82), and a mean Pi level > 2.5 mg/dL was associated with a better neurological outcome at ICU discharge (Figure 5).

## Discussion

This observational study included a cohort of patients with sSAH admitted to a Neuro-ICU and a group of nonneurocritical patients admitted to the same ICU to determine the association between phosphate variability and outcomes, mainly neurological outcomes.

We found that hypophosphatemia was more frequent in patients with sSAH. Patients with sSAH had increased odds of having hypophosphatemia at admission compared with nonneurocritical patients. Mean phosphate levels were also lower, despite a significantly higher percentage of days with EN. Similar results were demonstrated in patients with nontraumatic intracranial hemorrhage where hypophosphatemia was common and occurred in the early phase of ICU stay [7]. However, we did not find any study including patients with SAH and the influence of phosphate variability on their outcomes. Studies including patients with severe head injury demonstrated that this population is at risk of developing hypomagnesemia, hypophosphatemia, and hypokalemia. Polderman et al. suggested that hypophosphatemia could be associated with nasogastric suction, liver disease, sepsis, alcoholism, and administration of various drugs, such as P-binding antiacids, catecholamines, b-adrenergic agonists, and sodium bicarbonate [6]. They believed that it might also be related to refeeding hypophosphatemia after starvation for a period as short as 48 hours that can commonly occur in critically ill patients [6,18]. Lastly, they suggested that polyuria induced by brain injury, demonstrated by their results, is another factor that can worsen this condition [6].

By contrast, hyperphosphatemia was more frequent in the nonneurocritical group, and more than half of the patients had at least one episode of hyperphosphatemia. In the sSAH group, not only the mean Pi level but also the daily Pi levels were lower, mostly in the first four days. This finding and the highest incidence of hypophosphatemia could be explained by an intracranial hemorrhage-induced sympathetic storm and an intracellular shift of phosphate by catecholamines [7].

In the nonneurocritical group, patients with higher APACHE II and SAPS II scores had higher Pi levels. This finding coincides with the mortality results; patients who died had higher Pi levels. This association was not significant in the sSAH group, and higher scores were associated with lower Pi levels. Suzuki et al. found that although hypophosphatemia was more common in critically ill patients who died, this was not an independent risk factor for mortality or major morbidity after adjusting for illness severity [19]. Zazzo et al. also confirmed that hypophosphatemia is common in surgical intensive care patients and is associated with a high mortality rate [20].

Since hypophosphatemia was frequent in sSAH patients, analyzed and quantified intravenous phosphate substitution therapy was necessary. As expected, phosphate dose repletion was higher in sSAH patients, but there was no strict protocol for intravenous phosphate substitution therapy as we found in previous studies [7]. Repletion therapy was prescribed according to the physician's decision.

We found a positive correlation between mean Pi levels and GCS-ICUd in the sSAH group and a negative correlation in the nonneurocritical group. This finding means that lower Pi levels were associated with a poor neurological outcome at ICU discharge in sSAH patients and a better outcome in the nonneurocritical group. In the study group, patients who presented with hypophosphatemia had lower GOS-6M without statistical significance.

Using a multivariate analysis in our cohort of patients with sSAH, we confirmed previous results; we found the opposite in the nonneurocritical group. The mean Pi level was an independent predictor of neurological outcomes in sSAH patients because higher Pi levels were associated with a better GCS-ICUd. A possible explanation for these results is the association between Pi levels and 2,3-diphosphoglycerate production in red blood cells. Therefore, hypophosphatemia might decrease 2,3-diphosphoglycerate and shift the hemoglobin dissociation curve to the left decreasing oxygen release. This phenomenon may lead to impaired brain energy metabolism and brain injury [21].

Therefore, we tried to identify a Pi level cutoff point that could be used as a target to obtain better neurological outcomes. In our analysis, the cutoff was a mean Pi level > 2.5 mg/dL. Guiding therapy to maintain the Pi concentration above this value could be a good strategy. In our study, creatinine and the blood urea level, the need for Pi substitution, and pH were independently associated with the mean Pi level during ICU stay. This finding contradicts the study by Junttila et al. [7] that did not find any acid-base or blood gas status difference between normo- and hypo-phosphatemic patients with nontraumatic intracranial hemorrhage.

Although mortality was not significantly associated with Pi levels, patients with sSAH who died had lower Pi concentrations. This absence of statistical significance can be due to the reduced number of patients who died in this group. Similarly, there was a trend of increasing ICU-LOS at lower Pi levels that had no statistical significance. The opposite results were observed in the nonneurocritical group for both mortality and ICU-LOS.

These results are in contrast with those in previous studies that include nontraumatic intracranial hemorrhage, where ICU mortality, three-month and one-year mortality rates, and long-term functional outcomes were similar between normophosphatemic and hypophosphatemic patients [7].

Study limitations

This was a single-center study with a small sample size, and our findings may not be generalizable. To assess the impact of the results on outcomes, the sample size should have been larger, and the present study was a retrospective study with potential systematic error and bias. We did not register therapeutic interventions such as insulin, catecholamines, glucocorticoids, sodium bicarbonate, and antacids, which can affect phosphate levels and our results. We did register diuresis and fluid balance but did not register the urine phosphate concentration to assess urine phosphate losses better. There was no strict protocol for phosphate substitution therapy.

## Conclusions

Patients with sSAH had lower mean phosphate levels and required significantly higher daily Pi replacement to achieve normal values compared with nonneurological patients. The phosphate level was independently associated with the neurological status at ICU discharge in the sSAH group; therefore, patients may need cautious phosphate repletion. Maintaining a phosphate concentration above 2.5 mg/dL in sSAH patients could be a good strategy. Further studies are warranted to understand the role of phosphate in the pathophysiology of sSAH, namely regarding brain metabolism, kidney-brain links, and outcomes.
